# Gut microbiota-derived butyrate selectively interferes with growth of carbapenem-resistant *Escherichia coli* based on their resistance mechanism

**DOI:** 10.1080/19490976.2024.2397058

**Published:** 2024-09-18

**Authors:** Eva Happ, Kora Schulze, Zinia Afrin, Sabrina Woltemate, Pia Görner, Stefan Ziesing, Dirk Schlüter, Robert Geffers, Volker Winstel, Marius Vital

**Affiliations:** a Institute of Medical Microbiology and Hospital Epidemiology, Hannover Medical School, Hannover, Germany; b German Center for Infection Research (DZIF), Partner Site Hannover-Braunschweig, Hannover, Germany; c Genomics Research Group, Helmholtz Centre for Infection Research, Braunschweig, Germany; d Research Group Pathogenesis of Bacterial Infections; TWINCORE, Centre for Experimental and Clinical Infection Research, a joint venture between the Hannover Medical School and the Helmholtz Centre for Infection Research, Hannover, Germany

**Keywords:** Antibiotic resistance, butyrate, *Escherichia coli*, bacterial physiology, anaerobic cultivation, gene expression, gut microbiota

## Abstract

We investigated consequences of resistance acquisition in *Escherichia coli* clinical isolates during anaerobic (continuous culture) growth and examined their sensitivity to butyrate, a hallmark metabolite of healthy gut microbiota. Strains were stratified based on carrying either a carbapenemase (CARB) or displaying porin malfunctioning (POR). POR displayed markedly altered growth efficiencies, lower membrane stability and increased sensitivity to butyrate compared with CARB. Major differences in global gene expression between the two groups during anaerobic growth were revealed involving increased expression of alternative substrate influx routes, the stringent response and iron acquisition together with lower expression of various stress response systems in POR. Longitudinal analyses during butyrate wash-in showed common responses for all strains as well as specific features of POR that displayed strong initial “overshoot” reactions affecting various stress responses that balanced out over time. Results were partly reproduced in a mutant strain verifying porin deficiencies as the major underlying mechanism for results observed in clinical isolates. Furthermore, direct competition experiments confirmed butyrate as key for amplifying fitness disadvantages based on porin malfunctioning. Results provide new (molecular) insights into ecological consequences of resistance acquisition and can assist in developing measures to prevent colonization and infection based on the underlying resistance mechanism.

## Introduction

It was estimated that in 2019 alone 4.95 million deaths were associated with antimicrobial resistance (AMR) with 1.27 million directly attributable to bacterial AMR.^1^ Given that carbapenems are often among the last line treatment for infections, the rise of carbapenem-resistant *Enterobacteriaceae* (CRE) is of great concern and in 2017 the World Health Organization classified CRE as one of the top three classes of drug-resistant bacteria that urgently need the development of new antibiotics.^2^ While CRE prevalence was low before 2000, it strongly increased worldwide in the last decade that is exemplified by data from China, where the resistance rate increased by 60% from 2015 to 2020.^3^ Settings harboring vulnerable groups, such as hospitals, are of particular concern. In a global multicenter study involving 64 medical centers that consecutively collected *Enterobacterales*, the overall resistance rate of CRE in 2019 was 4.5%.^4^ For ICU patients, especially high burdens of CRE infections have been reported with mortalities up to 50%.^5^ The most abundant species comprising CRE are *Escherichia coli* and *Klebsiella pneumoniae*, where several mechanisms promoting resistance have been described. Most common are carbapenemases, antibiotic hydrolyzing enzymes that act in the periplasm, and certain structural alterations, mainly porin-deficiencies and increased expression of efflux pumps that cause decreased influx and increased efflux of the antibiotic, respectively.^6,7^ Expression of a carbapenemase is often sufficient to provide a full-resistant phenotype, whereas porin deficiencies require a combination with extended spectrum beta-lactamases (ESBL) to generate a full-resistant phenotype against carbapenems.^8,9^ Due to the fundamental difference of the two mechanisms, they cause greatly distinct physiological responses of respective bacteria during antibiotic challenge.^9^ Epidemiological surveys indicate that carbapenemase-based resistance phenotypes prevail worldwide; however, in Europe and the United States, other mechanisms are highly prevalent, especially in the case of *E. coli*.^10,11^

Apart from understanding mechanisms governing resistance phenotypes, elucidating the consequences of resistance acquisition for ecophysiology of CRE, such as growth performance and adaptation to stressful conditions, is a top priority as it bears the potential to develop precision measures based on the resistance mechanism limiting infection . In this context, preventing CRE colonization is an important preventive measure as it was described that 16.5% of patients carrying CRE also develop CRE-associated infections;^12^ for ICU patients, numbers up to 50% were reported.^5^ While the presence of carbapenemases is expected to affect general physiology of carriers at a minor level, structural alterations, especially porin deficiency, are known to be in a trade-off between higher stress resistance and less nutrient scavenging capabilities.^13^

In *E. coli*, the two major outer membrane porins (Omp) are OmpF and OmpC that are made of beta-barrel transmembrane proteins located in the outer membrane allowing permeation of small hydrophilic molecules, such as nutrients (e. g. glucose and amino acids) as well as beta-lactam antibiotics including carbapenems.^14,15^ Due to its larger diameter, OmpF is considered more important for influx of antibiotic molecules, while OmpC is additionally critical for membrane integrity and adaptation to envelope stress.^16,17^ OmpC mutant strains showed higher sensitivity to ethanol^16^ and bile salts^18^ indicating that the resistance mechanism also influences adaptation capabilities to gut microbiota-derived metabolites. Using animal models, it was shown that gut microbiota maintain colonization resistance against (antibiotic resistant) pathogens via various mechanisms including nutrient competition and the production of metabolites, such as short-chain-fatty acids (SCFAs) and secondary bile acids.^19,20^ However, the specific role of the resistance mechanism was not investigated in this context. SCFAs, primarily acetate, propionate, and butyrate, are produced by gut microbiota reaching physiological concentrations between 20 and 140 mM, where highest concentrations are found in the proximal colon continuously decreasing toward the distal colon.^21^ Acetate is produced by most members of gut microbiota, whereas propionate is primarily synthesized by several members of the *Bacteroidetes;* specific taxa of the *Firmicutes* are major butyrate producers.^22^ Their action is frequently explained by diffusion of the protonated form through the outer membrane subsequently causing proton pressure in the periplasm by dissociating.^20,23^ Given that the internal pH of *E. coli* is usually maintained at slightly alkaline levels, acidification through SCFAs causes severe stress for the bacterium.^20^ Next to this intracellular acidification, SCFAs are known to intercalate in the inner membrane after entering the periplasm causing higher membrane fluidity.^19^ How the antibiotic resistance mechanism, in particular dysfunctions of Omps, influences adaptation and responses to physiological concentrations of SCFAs is currently unknown. To this end, the aim of this study was to investigate the response of carbapenem-resistant *E. coli* to butyrate on a molecular level under anaerobic conditions, specifically focusing on porin-deficient strains in comparison to carbapenemase-carrying bacteria.

## Materials and Methods

### Bacterial strains and culture conditions

Bacterial strains were isolated from patients between 2013 and 2019 at the Institute of Medical Microbiology and Hospital Epidemiology of Hannover Medical School, Germany, and screened *in silico* for their resistance mechanism based on Illumina sequencing as described in our previous study.^9^ In brief, DNA was extracted (DNeasy PowerSoil Pro Kit, Qiagen, Germany) and libraries were prepared (Illumina DNA Prep, Illumina, United States) and subsequently sequenced on Illumina NovaSeq 6000 (at Helmholtz Centre for Infection Research (HZI)) in paired-end mode (2 × 150 bp) to achieve a coverage >50×. Reads were quality filtered via fastp (v0.19.5, *default mode*) and assembled using SPAdes (v3.15.5, *careful mode*).^24^ Subsequent gene calling was performed with prokka (v1.14.6, *-fast mode*)^25^ and the Average Nucleotide Identity (ANI) was calculated using fastANI (v1.33, *default mode*).^26^ A dendrogram was constructed in R (4.2.2) with dendextend (v1.17.1). Determination of genes encoding carbapenemases and malfunctioning porins was done using ariba (v2.14.4, *default mode*) based on the CARD database (version July 2023).^27^ We randomly selected 15 strains exhibiting a carbapenemase (either OXA-48, NDM or VIM) with intact porins and 15 strains characterized by alterations (deletions or interruptions) of *ompC*, *ompF* genes without carrying a carbapenemase (Figure S1). Pan/core-genome analyses were done based on the program roary (v3.13.0, *default mode*).^28^ Single nucleotide polymorphism (SNP) analysis was performed via snippy (v4.6.0) on the contig level taking *E. coli* ATCC 8739 as the reference. For Ertapenem, minimal inhibitory concentrations (MIC) based on VITEK 2 were above the breakpoint of 0.5 mg L^−1^ (according to EUCAST) for all strains, whereas those for Meropenem were sometimes below the breakpoint of 8 mg L^−1^. All experiments were approved by the local ethics committee (#9399_BO_K_2020).

### Batch cultures

For all anaerobic experiments, a minimal medium according to Schäfer et al.^9^ was used that is based on reference^29^ and contained 1 g L^−1^ Glucose, 0.5 g L^−1^ casamino acids and 5 mL L^−1^ trace elements (TE) consisting of 13.42 g MgCl_2_*6 H_2_O L^−1^, 8 g CaCO_3_ L^−1^, 7.74 g FeCl_3_*6 H_2_O L^−1^, 1.15 g MnCl_2_*4 H_2_O L^−1^, 0.146 g CuSO_4_*5 H_2_O L^−1^, 0.13 g CoCl_2_*6 H_2_O L^−1^, 0.4 g ZnO L^−1^, 1.24 g H_3_BO_3_ L^−1^, 1.04 g NaMoO_4_*2 H_2_O L^−1^, 2 g NiCl_2_*6 H_2_O L^−1^, 0.84 g SeO_3_ L^−1^ and 87.4 g EDTA Na_4_*2 H_2_O L^−1^ (equimolar to di- and trivalent cations). The medium (excluding glucose and TE) was autoclaved, subsequently boiled to remove all oxygen and cooled-down under continuous nitrogen overgasing to sustain anaerobic conditions while adding the indicator resazurin (0.0017 g L^−1^) as well as cysteine (0.5 g L^−1^). Before use sterile-filtered glucose and autoclaved TE were added. For screening experiments, bacteria from anaerobic over-night cultures (ONC; V = 3 mL) incubated at 37°C in an anaerobic chamber (Coy Laboratory Products, Grass Lake, MI, USA; fed by N_2_ and an anaerobic gas-mixture consisting of 10% CO_2_, 10% H_2_ and 80% N_2_) were inoculated into deep-well plates (Sarstedt, Germany) (V = 1.2 mL) at a starting OD of 0.01 containing 0, 25, 50 and 75 mM sodium butyrate (Roth, Germany). Plates were subsequently incubated at 37°C in the anaerobic chamber. The OD (600 nm) was measured by a plate-reader (SYNERGY HTX Multi Mode Reader, BioTek, United States) transferring 200 μL of each culture after 18 h, 24 h, and 48 h into clear well plates (Greiner Bio-One, Austria); recorded OD values for this high-throughput method were, hence, lower compared with standard OD measurements that are usually based on single cuvettes exhibiting a width of 10 mm. Experiments were performed in replicate samples (*n* = 2). Cultures for determining growth curves were prepared in hungate tubes (V = 5 mL) containing 0 mM, 6.25 mM, 12.5 mM, 25 mM, and 50 mM sodium butyrate (Roth, Germany), incubated at 37°C (200 rpm) and the OD (600 nm) was measured on a Genesys 20 instrument (Thermo Fischer Scientific, United States) each hour for 8 h on replicate samples. Area Under the Curve (AUC) values were determined using the function *auc* from the gcplyr (v1.10.0) package.

### Continuous culture experiments

Continuous culturing was performed as described previously based on self-assembled mini bioreactors^9,30^ using the anaerobic minimal media as described above. The whole system, including the feed medium, was kept under anaerobic conditions throughout the experiments by constant nitrogen overgasing of reactors and the feeding bottle. Anaerobic ONCs (6 mL) were inoculated into reactors (100 mL) and grown in batch mode for 5 h until bacteria reached late exponential phase. Continuous culturing at a dilution rate of 0.2 h^−1^ was initiated by starting the peristaltic pump and after 5–10 volume changes (1–2 days) strains reached steady state. Samples (1 mL; after discarding the first 2 mL that represent cultures in sampling port) were taken every 30 min for OD measurements, where 0.5 mL were transferred into a plastic cuvette and subsequently measured on a Genesys 20 instrument (Thermo Fischer Scientific, United States). Samples for RNASeq analyses (1 mL) were taken and directly transferred to 1 mL RNA later (Thermo Fisher Scientific, United States), spun down (13.000 rpm) for 10 min at 4°C and the pellet was resuspended in 300 μL RNAlater before storage at −80°C as described previously.^9^ For glucose measurements, supernatants of samples (13.000 rpm for 10 min at 4°C) were enzymatically analyzed (Glucose-Glo Assay, Promega, United States) according to the manufacturer measuring luminescence with a plate-reader (SYNERGY HTX Multi Mode Reader, BioTek, United States). After steady-state measurements, butyrate was added to the feed medium at 50 mM final concentration starting the wash-in phase. Butyrate concentrations in reactors were calculated based on wash-in kinetics of substrate in continuous culture (s_t_=s_in_*(1-e^D*t^), where s_in_ = 50 mM and D (dilution rate) = 0.2). Samples for monitoring growth (OD) were taken every 30 min during the experiment, and samples for residual glucose (G1, G2, G3) and for RNASeq (R1, R2, R3) were taken at steady state and two time-points during the wash-in phase (calculated concentration of butyrate was 25 mM and 43 mM, respectively). All experiments were performed in replicate (*n* = 2) samples.

### Experiments based on a porin mutant strain

The gut-derived *E. coli* ATCC 8739 was used to create a *∆ompC/∆ompF-* double mutant strain, and gene disruption was essentially performed as described before.^31^ In brief, *ompC*- or *ompF*-targeting PCR products containing a chloramphenicol resistance marker were generated using the primers listed in Table S1 and pKD3 as a template. The resulting PCR products were gel-purified, digested with *Dpn*1, and subsequently re-purified. Next, chemically competent *E. coli* were transformed with the λ Red recombinase expression plasmid pKD46 according to standard laboratory protocols. Transformants harboring pKD46 were then grown at 30°C in LB medium containing ampicillin and L-arabinose (10 mM) to a final OD of 0.5, harvested via centrifugation, and made electrocompetent using ice-cold glycerol solution (10%). Electrocompetent cells were transformed with *ompC*- or *ompF*-targeting PCR products using a BioRad GenePulser apparatus following the manufacturer’s instructions. Immediately after the pulse, cells were carefully mixed with 900 µl of LB medium, incubated at 37°C for 90 min, and plated onto selective media containing chloramphenicol. Following selection, transformants were colony-purified on nonselective media and analyzed for helper plasmid loss via ampicillin-sensitivity testing. Sanger sequencing was used to verify replacement of the chromosomal target region with the chloramphenicol resistance marker. Finally, the resistance marker was eliminated in sequencing-validated clones using the pCP20 system as described elsewhere.^31^ The resulting mutant candidate was verified via PCR and whole-genome sequencing. Batch and continuous culture experiments were performed in replicate samples with the *∆ompC/∆ompF* double mutant (mt) and wildtype (wt) strain based on the same procedures as described above for clinical isolates.

For competition experiments, wt and mt were cultured in hungate tubes (37°C, 200 rpm) containing minimal media with 0 mM or 50 mM sodium butyrate (Roth, Germany), respectively, as described above. Cells of both cultures were harvested at exponential growth phase and combined at equal ratios in fresh media reaching a combined starting OD of 0.05. Cultures were subsequently incubated at 37°C and samples were taken immediately (T0) and at beginning of stationary phase (T1).

### Experiments investigating membrane stability

Experiments on agar plates were done similar to Choi & Lee (2019).^16^ Strains were grown over night at 37°C under anaerobic conditions and serial tenfold dilutions were prepared in 1 × PBS (pH = 7.2). Subsequently, 5 μL of the 10^−3^ − 10^−7^ dilutions were spotted in replicate samples (*n* = 2) on LB agar plates and on plates additionally containing 2% SDS. All experiments were performed under anaerobic conditions in an anaerobic chamber and plates were incubated at 37°C over night. On the next day, pictures of agar plates were taken using a GelDoc XR+ device (Bio-Rad Laboratories, United States). For Figure 7, pictures’ brightness and contrast were adjusted using power point.

Strains were additionally challenged with SDS in liquid culture and the uptake of the membrane impermeable dye SYTOX green (5 mM in DMSO; Thermo Fisher Scientific, United States) was monitored over time. ONCs were diluted 100 × in 1 × PBS (ph = 7.2; V = 1 mL) and SDS (0.025% final concentration) was added along with SYTOX green (final concentration of 0.75 μM). Solutions were incubated at room temperature and aliquots were diluted tenfold in 1 × PBS (after 5, 10, 15 and 25 min) before measuring on a CYTEK Northern Lights flow cytometer (Cytek Biosciences, United States). Events were recorded on a sideward-scatter (SSC) green fluorescence (B1) biplot. To get total concentration of cells, solutions were stained after 25 min with EDTA (5 mM final concentration) and SYBR Green I (Thermo Fisher Scientific, United States; 10000 × diluted final concentration) for an additional 15 min as described previously^9^ and events were recorded on the same plot as for SYTOX green above.

### RNA extraction, bioinformatics and data analysis

RNA extraction and library preparation were done as described previously.^9^ In brief, RNA was extracted (RNeasy Mini Kit, Qiagen, Germany) followed by DNase I treatment (RNase-Free DNase Set, Qiagen, Germany). Ribosomal RNAs were depleted by the NEBNext Bacterial rRNA Depletion Kit (NEB, United States), libraries were generated using the NEBNext Ultra II Directional RNA Library Prep Kit (NEB, United States) and subsequently sequenced on an Illumina NovaSeq 6000 (at HZI) in paired-end mode (2 × 150 bp; ∼5 × 10^6^ reads per sample).

For strains used in RNASeq analyses, assembled genomes (spades output from above) were uploaded to the RAST server that provides gene calling and annotations based on SEED’s Subsystems Technology.^32^ Individual RAST groupings are highlighted as italic in the text. Raw reads from RNASeq were analyzed in R (v4.2.2) as done previously^9^ involving quality filtering and depletion of sequences derived from rRNA (custom database based on our genomes) using KneadData (Huttenhower lab; v0.7.2) and subsequent mapping to references using BBMap (from JGI, v38.22; paired-end mode). Transcript coverage files were calculated (pileup.sh script from BBMap) and loaded into R to calculate Transcripts Per Million (TPM) and fold changes between conditions for all strains (function *foldchange* form the package gtools (v3.9.4)). Statistics were based on DEseq2 (v1.32.0) analysis on raw count data for all genes shared between the six isolates and on all RAST levels taking an adjusted *p*-value of 0.1 as cutoff, as suggested by the developers.^33^ Non-metric multidimensional scaling analysis was performed on TPM data of shared genes based on Bray-Curtis dissimilarities (function *metaMDS* (distance = “bray”, autotransform = TRUE) using the package vegan (v2.5.7)) and visualized via ggplot2 (v3.3.5). Samples taken during competition were subjected to Illumina sequencing as described above and proportion of strains were calculated from average coverage pattern of *ompC* and *ompF* compared with average coverage pattern of all other genes based on BBMap outputs. Other figures were generated by GraphPad Prism (v.9.0.0) and Biorender. For results, the mean and standard deviations are given and a Two-Way ANOVA (function *aov*) was used for statistical analyses including the resistance mechanism and butyrate as independent variables, as well as their interaction, with Tuckey HSD post-hoc testing (function *TukeyHSD*). Repeated measures ANOVA (function *anova_test*) from the package *rstatix* (v0.7.2) for each group including paired student’s t-test post-hoc testing (function *pairwise_t_test*) was additionally performed for those results.

All bioinformatics analyses were performed on MHH’s High-Performance Computer Cluster (HPC).

## Results

The aim of the study was to investigate the growth physiology of carbapenem-resistant *E. coli* strains under anaerobic conditions and elucidate their adaptation mechanisms to butyrate, specifically focusing on uncovering the role of the resistance mechanism in this context. Based on genomic analyses, the 15 strains exhibiting a carbapenemase (CARB), either OXA-48, NDM or VIM, harbored intact *ompC* and *ompF* genes, whereas the other 15 strains showed alterations in *omp* genes (POR) and did not encode any carbapenemase (Figure S1). Additionally, all strains carried ESBL-genes (except for one CARB strain). MIC values for Ertapenem ranged from 0.5 mg L^−1^ to 8 mg L^−1^ (CARB) and 2 mg L^−1^ to 8 mg L^−1^ (POR), whereas for Meropenem values ranged from 0.125 mg L^−1^ to 16 mg L^−1^ (CARB) and 2 mg L^−1^ to 16 mg L^−1^ (POR). No phylogenetic clustering of strains based on the resistance mechanism was observed and detailed comparative genome analyses did not identify any genes or SNPs specific for either group suggesting that porin deficiency was indeed the only major genomic discriminator between POR and CARB (Figure S2).

### POR and CARB strains differed in final yields and growth dynamics during butyrate challenge in batch culture

**Figure 1.**
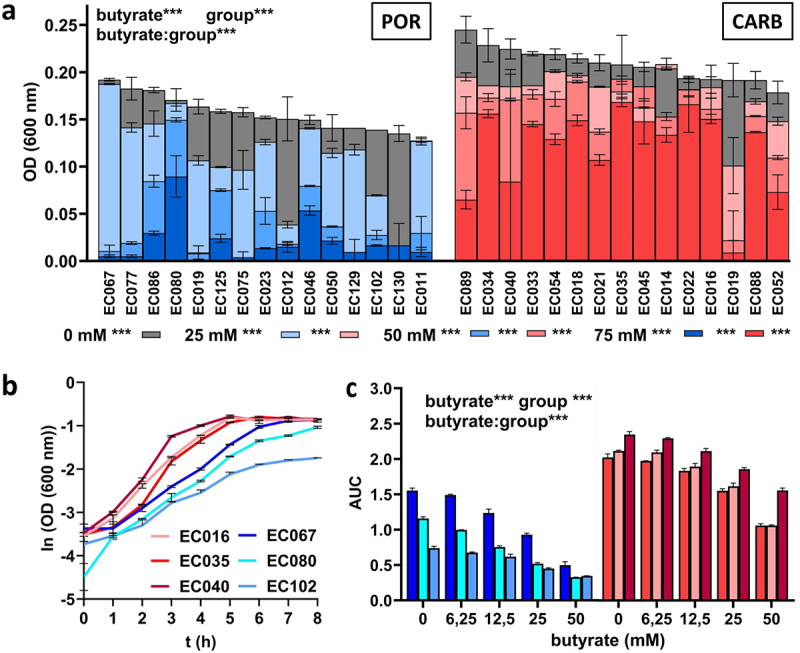
Batch growth of POR (blue) and CARB (red) strains at different concentrations of butyrate (0 mM, 25 mM, 50 mM and 75 mM) under anaerobic conditions. Final growth (OD 600) after 48 h (*n* = 15 strains for each group) are shown in panel a, whereas growth curves of three selected POR and CARB strains at 0 mM butyrate are given in panel b. The influence of butyrate on growth dynamics based on the area under the curve (AUC) calculations from growth curves for the selected strains is given in panel c. All individual growth curves are given in Figure S3. The mean of replicate samples (*n* = 2) is given, where error bars display standard deviations. Statistical calculations are based on two-way ANOVA analyses with Tukey’s HSD post-hoc testing, where “butyrate” refers to the effect of butyrate, “group” describes the effect of the resistance mechanism and “butyrate:group” gives their interaction. *, **, ***; *p* < .05, *p* < .01, *p* < .001. Statistics of post-hoc testing in panel a are given as follows: results right of the butyrate concentration signify difference between groups, whereas comparisons of each concentration to 0 mM butyrate within each group is given next to color-coded boxes.

We cultured all POR and CARB strains in anaerobic batch culture under a range of butyrate concentrations (0 mM−75 mM) and monitored their growth based on optical density (OD) measurements. Significant differences in final growth between POR (0.154 ± 0.020) and CARB (0.203; ±0.030) were observed at 0 mM butyrate (Figure 1(a)). Final growth was significantly lower in strains of both groups in the presence of butyrate at all concentrations in a dose-dependent manner. Overall, POR showed higher sensitivity to butyrate, where most strains barely grew at the two highest concentrations (0.037 ± 0.042 (50 mM) and 0.020 ± 0.023 (75 mM)), whereas CARB strains were still able to reach final ODs of 0.151 ± 0.047 and 0.122 ± 0.049 in the presence of 50 mM and 75 mM butyrate, respectively. With a few exceptions, similar results for all strains within a group were recorded.

Next, we investigated the effect of butyrate on growth dynamics. Three strains of each resistance group EC076, EC080, EC102 (POR) and EC016, EC035, EC040 (CARB) were cultured in hungate tubes with different concentrations of butyrate (0 mM−50 mM) and area under the curve (AUC) values from growth curves were calculated. Those representative strains were randomly selected covering the phylogenetic breath of all 50 strains and avoiding phylogenetic clustering of strains according to the resistance mechanism. Similar to observations of final growth yields, differences in AUC between POR and CARB strains were observed without butyrate exposure (Figure 1(b)), where POR (1.15 ± 0.36) displayed significantly reduced values compared with CARB (2.17 ± 0.15) (Figure 1(c)). In both groups, growth dynamics were unaffected up to a concentration of 6.25 mM butyrate and started to decline at 12.5 mM butyrate, where the effect of the metabolite was significantly stronger (*p* < .01) in POR compared with CARB. At 50 mM, butyrate AUC values for POR and CARB were 35.69% ± 9.75% and 56.29% ± 8.81%, respectively, compared with results during growth with 0 mM butyrate (Figure 1(c), S3).

### Growth of POR and CARB in continuous culture and their responses to butyrate over time

**Figure 2.**
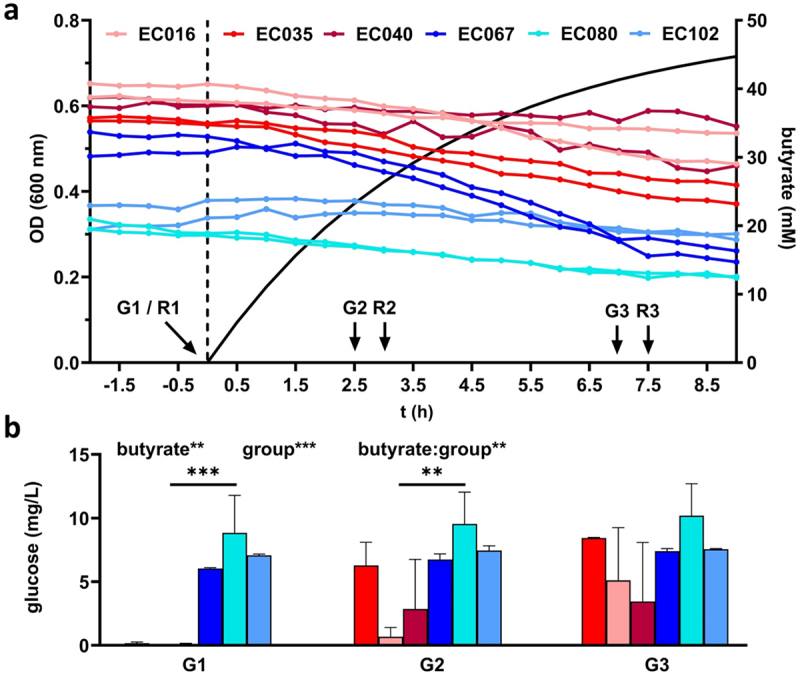
Continuous culture growth of three POR (blue) and three CARB (red) strains under anaerobic conditions. Panel a gives the optical density of cultures in steady state and during butyrate wash-in (separated by the dashed line; calculated butyrate concentration is shown as solid black line). Sampling points for RNASeq (R1-R3) and glucose measurements (G1-G3) are indicated. The amount of residual glucose concentrations in reactors is displayed in panel b (the mean and standard deviation are given based on replicate (*n* = 2) measurements for each culture). Statistical calculations are based on two-way ANOVA analyses with Tukey’s HSD post-hoc testing, where “butyrate” refers to the effect of butyrate, “group” describes the effect of the resistance mechanism and “butyrate:group” gives their interaction. *, **, ***; *p* < .05, *p* < .01, *p* < .001.

To investigate adaptation of POR and CARB to butyrate on a molecular level, we performed continuous culturing of the six selected strains challenging them with increasing concentrations of butyrate (0 mM−50 mM) over time and monitored their responses using RNASeq analyses. Given the distinct growth characteristics of strains and their responses to butyrate, continuous culturing was selected as the method of choice in order to exclude any confounding effects, especially differences in growth rates, on physiology and underlying molecular mechanisms. In steady state without butyrate, POR showed significant lower ODs (0.389 ± 0.107) than CARB (0.596 ± 0.036) (Figure 2(a)). Residual glucose concentrations were very low in all reactors confirming carbon limited conditions, however, POR strains displayed significant higher concentrations (7.312 ± 1.419 mg L^−1^) than CARB (0.1033 ± 0.063 mg L^−1^) (Figure 2(b)). During wash-in of butyrate residual glucose remained at low levels; a significant increase for CARB strains was observed from 0.103 ± 0.063 mg L^−1^ (0 mM) to 5.655 ± 2.538 mg L^−1^ (43 mM) over time, while concentrations in POR remained at similar levels (Figure 2(b)). Despite carbon limited conditions throughout the experiment, a decrease in OD was observed in all cultures with strain-specific responses; no distinct patterns between POR and CARB were detected (Figure 2(a)). Monitoring of pH verified that all cultures grew at mildly acidic conditions. Values were constant throughout the experiment for each culture and only minor differences between strains, with on average slightly lower values in CARB, were observed (Table S2).

### Overall gene expression patterns between POR and CARB in continuous culture

**Figure 3.**
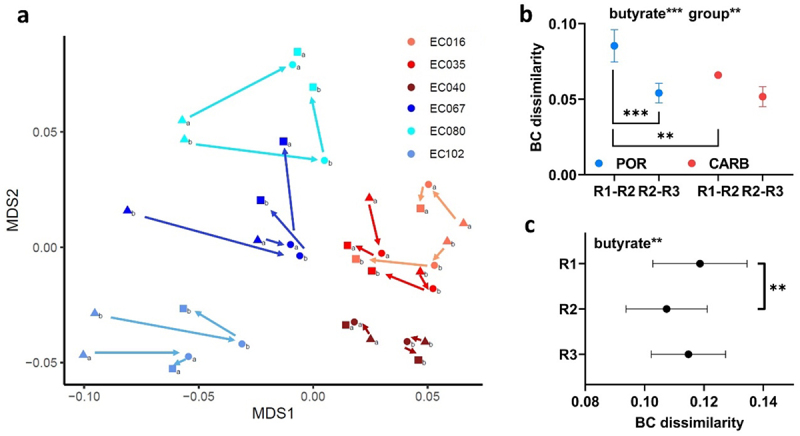
Gene-expression analysis of POR (blue) and CARB (red) strains during anaerobic continuous culture growth in steady state and during butyrate wash-in (two time-points). In panel a ordination analysis based on metric multidimensional scaling (MDS) analysis and Bray Curtis (BC) dissimilarity is given; results for steady state (0 mM butyrate; triangle; R1) and at butyrate concentrations of 25 mM (dot; R2) and 43 mM (square; R3), respectively, are displayed. BC dissimilarities between R1 and R2 and for R2 to R3 within strains of the two groups are shown in panel b, whereas dissimilarities between POR and CARB strains of the three time-points are given in panel c. The mean and standard deviation are shown. Statistical calculations are based on two-way ANOVA analyses with Tukey’s HSD post-hoc testing where “butyrate” refers to the effect of butyrate and “group” describes the effect of the resistance mechanism. *, **, ***; *p* < .05, *p* < .01, *p* < .001.

Samples for transcriptome analyses were taken at steady state (R1; 0 mM butyrate) to compare growth physiologies of strains of the two groups under anaerobic carbon-limited conditions. Furthermore, samples were taken at R2 (25 mM) and R3 (43 mM) to monitor molecular adaptation strategies of POR and CARB to butyrate over time. For analyses, all genes shared between all strains (*n* = 3,277) were considered. At steady state, POR and CARB strains clustered separately in ordination analysis demonstrating that the resistance mechanism *per se* governed overall gene expression that was maintained throughout the experiment (Figure 3(a)). Upon butyrate challenge expression patterns changed in both POR and CARB, where POR displayed significant greater shifts at the beginning of the butyrate wash-in (R1 to R2; based on Bray-Curtis dissimilarity (BCdis)) than CARB, whereas between the two following timepoints (R2 to R3) similar magnitudes in BCdis were detected in POR and CARB (Figure 3(b)). Moreover, gene expression differences between the two groups significantly declined at 25 mM butyrate (from BCdis 0.119 ± 0.016 to BCdis 0.107 ± 0.014) (Figure 3(c)) clustering samples closer together in ordination analyses at R2 compared with R1 (Figure 3(a)). Gene expression patterns between POR and CARB diverged again at R3 (Figure 3(c)).

### Growth in continuous culture at steady state (0 mM butyrate) – features distinct between POR and CARB

**Figure 4.**
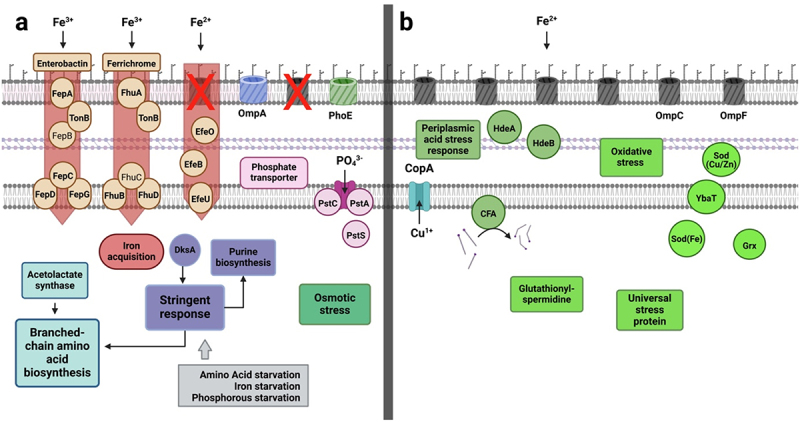
Mechanistic model based on significantly differentially expressed genes and RAST subsystems in POR and CARB during steady state growth (0 mM butyrate). On the left (panel a) features higher expressed in POR are shown, whereas those expressed at higher levels in CARB are shown on the right (panel b). Genes encoding unspecific porins of the outer membrane along with several iron uptake systems, the stringent response and synthesis of branched chain amino acids as well as the osmotic stress response were main features higher expressed in POR, whereas several other stress response systems and a copper stress sensing protein showed higher levels in CARB. Cycloproprane-fatty acyl phospholipid synthase (cfa) is displayed with its main function to catalyze integration of cyclopropane ring in membrane fatty acids. RAST subsystems are given as boxes (except for iron acquisition belonging to a RAST category) and single proteins are shown as circles. FepB and FhuC (standard font) were not significantly differentially expressed and are shown for comprehensive reasons. For detailed explanations see the text and Tables S3 and S4(a).

To elucidate growth differences of strains of the two groups on a molecular level in detail, we first focused on steady-state growth without butyrate (0 mM). A summary of differentially expressed features is given in Figure 4 (Table S3) covering those considered most important; a detailed list including all genes is given in Table S4(a). We found genes coding for unspecific porins of the outer membrane higher expressed in POR than CARB, namely *ompA* and *phoE* with a log2foldchange (lg2fc) of 3.123 and 7.150, respectively. Excessively high expression of the latter was mainly driven by strain EC102, while *phoE* expression of the other POR strains displayed similar levels as CARB strains. Next, the subsystem *Stringent response* (lg2fc: 0.350) and its inductor *dksA* (lg2fc: 0.600) were higher expressed in POR at steady state along with the category *Iron acquisition and metabolism* (lg2fc: 1.297) (Figure 4(a)). The latter was mainly driven by the uptake systems *fepABCDG* and *fhuABCD* based on siderophores, namely, enterobactin and ferrichrome, respectively, acting on ferric iron (Fe^3+^), and *efeOUB* that catalyzes uptake of ferrous iron (Fe^2+^), which is specifically relevant under anaerobic conditions. Furthermore, biosynthesis of several amino acids, mainly the subsystem *Branched-chain amino acids* (Valine, Leucine, Isoleucine) (lg2fc: 1.331) together with its precursor the subsystem *Acetolactate synthase* (lg2fc: 1.514), as well as genes for the subsystem *De-novo Purine biosynthesis* (lg2fc: 0.351) displayed higher expression levels in POR than CARB at steady state (Figure 4(a)). The subsystem *High affinity phosphate transporter* (*pst*; lg2fc: 1.190) was also higher expressed in POR, which was, however, mainly driven by values form EC102 (Table S3).

The subcategory *Osmotic stress* (lg2fc: 0.254) was slightly higher expressed in POR than CARB at steady state, whereas the majority of genes connected to various other stress responses was higher expressed in CARB (Figure 4(b)). In particular, the periplasmic chaperones *hdeA* (lg2fc: 3.732) and *hdeB* (lg2fc: 4.452), which are part of the subsystem *Periplasmic acid stress response* (lg2fc: 3.895), were higher expressed in CARB along with the gene for *Cycloproprane-fatty acyl phospholipid synthase* (*cfa*; lg2fc: 1.955). Moreover, enzymes connected to the subsystem *Oxidative stress* (lg2fc: 0.251), namely genes for superoxide dismutase (*sod(Fe);* lg2fc: 1.795), *sod(Cu/Zn);* lg2fc: 1.314), subsystem *Glutaredoxin (Grx;* lg2fc: 0.770) and the copper stress sensing transmembrane protein *ybaT* (lg2fc: 2.709), as well as the subsystem *Universal stress protein family* (lg2fc: 0.908) and subsystem *Glutathionylspermidine and Trypanothione* (lg2fc: 0.456) were significantly higher expressed in CARB (Figure 4(b)). The copper translocating enzyme (*copA*) was also higher expressed in strains of that group (lg2fc: 1.528).

### Response to butyrate in continuous culture – features common to all strains

**Figure 5.**
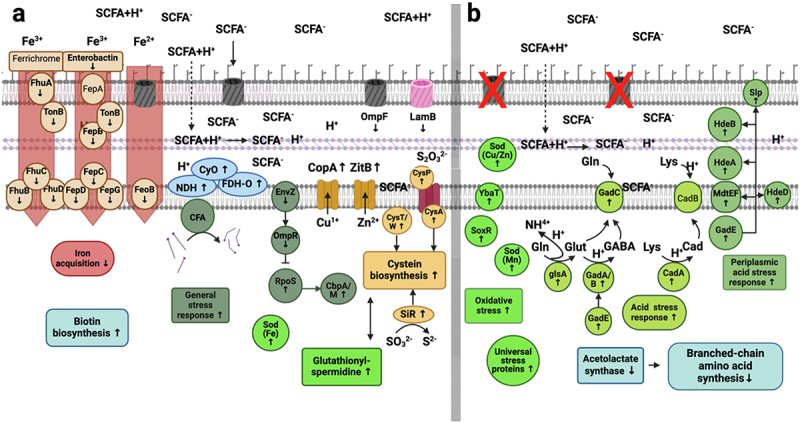
Mechanistic model of significantly differentially regulated genes and RAST subsystems during butyrate challenge in comparison to steady state (up-/down regulation is indicated by arrows). Regulated features common to both groups (POR+CARB) are shown on the left (a), whereas features specific for POR are displayed on the right (b). Common responses comprised a downregulation of *lamB* and *ompF* genes associated with iron uptake, whereas the general stress response, genes encoding enzymes involved in cysteine and biotin synthesis as well as those encoding specific metal transporters were upregulated. Strains of the POR group additionally increased expression of several stress response genes primarily connected to acid stress response, whereas those involved in branched-chain amino acid synthesis were downregulated. RAST subsystems are given as boxes (except for iron acquisition that represents a RAST category; acid and general stress responses are not specifically annotated in RAST and were manually categorized), single proteins are shown as circles. Effects of deprotonated butyrate (SCFA^−^), which can enter the periplasm through porins and intercalate in the inner membrane, as well as its protonated form (SCFA+H^+^), which can cause acidic stress in the periplasm by diffusion through the outer membrane subsequently releasing protons, are indicated. Cycloproprane-fatty acyl phospholipid synthase (cfa) is displayed with its main function to catalyze integration of cyclopropane ring in membrane fatty acids. The siderophore ferrichrome, FepA and CadB (standard font) are shown for completeness, despite not being significantly regulated in either group. For detailed explanation see the text and Tables S4(b, c) and S5.

Next, we looked at features of all strains that governed responses to butyrate (R2 (25 mM) and R3 (43 mM)) compared with R1 (0 mM) irrespective of the resistance mechanism. A summary of features we considered most important is given in Figure 5 (Table S5); for a detailed list of all genes see Table S4(b,c). Consistent with ordination analysis that clustered all strains closer together upon initial butyrate challenge (Figure 3(a)) many genes and pathways were similarly affected in both POR and CARB strains indicating a universal response to butyrate (Figure 5(a)). It involved downregulation of maltoporin gene (*lamB*; lg2fc POR: 0.472/lg2fc CARB: 3.214) along with upregulation of genes encoding for the general stress response. For instance, sigma factor S (*rpoS*; lg2fc POR: 0.759/lg2fc CARB: 0.134) and its related chaperone *cbpA* (lg2fc POR: 1.221/lg2fc CARB: 0.160) were increasingly expressed with butyrate in strains of both groups, while its inhibiting factors *envZ* (lg2fc POR: 0.656/lg2fc CARB: 0.301) and *ompR* (lg2fc POR: 0.587/lg2fc CARB: 0.372) were downregulated. Parts of the oxidative stress response, namely the subsystem *Glutathionylspermidine and Tryptathione* (lg2fc POR: 0.838/lg2fc CARB: 0.626) and superoxide dismutase (*sod (Fe*)) (lg2fc POR: 0.685/lg2fc CARB: 0.845) were also higher expressed. Other genes upregulated involved those coding for the subsystem *Cysteine biosynthesis* (lg2fc POR: 0.725/lg2fc CARB: NS), namely, *cysT* (lg2fc POR: 1.717/lg2fc CARB: 1.559) and *cysW* (lg2fc POR: 1.628/lg2fc CARB: 1.523), as well as genes encoding the thiosulfate uptake transporter *(cysP/A*; lg2fc POR: 2.228/lg2fc CARB: 1.394) and the sulfite reductase (*siR*; lg2fc POR: 2.000/lg2fc CARB: 1.575). Furthermore, the category *Iron acquisition and metabolism* (lg2fc POR: 1.314/lg2fc CARB: 2.100) was downregulated including iron acquisition pathways acting on ferric iron based on siderophores, namely, *fepABCDG* and *fhuABCD* along with the ferrous iron transporter *feoB* in both groups (Figure 5(a)).

Additionally, upregulation of a copper transporter (*copA;* lg2fc POR: 1.257/lg2fc CARB: 0.596) and a zinc efflux transporter (*zitB;* lg2fc POR: 1.001/lg2fc CARB: 0.587) as well as the gene for *Cycloproprane-fatty acyl phospholipid synthase* (*cfa*; lg2fc POR: 1.137/lg2fc CARB: 0.319) and genes encoding the subsystem *Biotin biosynthesis* (lg2fc POR: 1.500/lg2fc CARB: 1.466) were observed in both groups as a response to butyrate challenge. Genes encoding enzymes of the respiratory chain (*Cytochrome-O ubiquinol oxidase* (*cyO*; lg2fc POR: 1.077/lg2fc CARB: 1.068), *NADH: ubiquinone oxidoreductase* (*NDH*; lg2fc POR: 0.486/lg2fc CARB: 0.499) and *Formate dehydrogenase-O* (*FDH-O*; lg2fc POR: 1.744/lg2fc CARB: 1.120) were upregulated as well (Figure 5(a)).

### Response to butyrate in continuous culture – features specific for POR strains

Genes whose expression exclusively changed in POR to butyrate challenge are shown in Figure 5(b) and were mostly linked to stress responses involving several genes of the subsystem *Periplasmic acid stress response* (regulated by *gadE* (lg2fc: 1.359)), namely, genes encoding the periplasmic chaperones *hdeA* (lg2fc: 1,638) and *hdeB* (lg2fc: 1.590), the starvation lipoprotein (*slp*; lg2fc: 1.535) of the outer membrane and *mdtEF* (lg2fc: 0.709), a putative multidrug efflux pump, along with *hdeD* (lg2fc: 1.575) (Figure 5(b)). Furthermore, genes associated with the intracellular acid stress response were exclusively upregulated in POR encompassing glutaminase *glsA* (lg2fc: 1.678) and glutamate decarboxylase (*gadA/B;* lg2fc: 2.547) as well as the connected antiporter *gadC* (lg2fc: 2.502), which enables bacteria to bind protons to glutamine or glutamate forming GABA that is exported in exchange for a new glutamine molecule. Parts of a similar system using lysine to form cadaverine by binding a proton were also found upregulated only in POR during butyrate challenge (*cadA;* lg2fc: 0.738). The gene of cadaverine/lysine antiporter *cadB* was, however, not affected during the wash-in of butyrate. Next to acid stress, the subsystem *Universal stress protein family* (lg2fc: 0.449) and parts of the subsystem *Oxidative stress response* (lg2fc: 0.436), such as the oxidative stress regulator *soxR* (lg2fc: 0.888) and two superoxide dismutases (*sod (Mn);* lg2fc: 1.011 and *sod (Cu/Zn*); lg2fc: 0.612) together with *ybaT* (lg2fc: 1.561), a protein connected to copper stress, were specifically upregulated in POR upon butyrate challenge. Genes encoding enzymes involved in the synthesis of certain amino acids, in particular the subsystem *Branched-chain amino acids* (lg2fc: 0.903), as well as the associated subsystem *Acetolactate synthase* (lg2fc: 0.908) were downregulated in POR (Figure 5(b)).

Gene expression changes signifying specific responses of CARB to butyrate were only a few involving a downregulation of *ompF* (lg2fc: 1.471) and *ompC* (lg2fc: 3.278) and a slight upregulation of the subsystem *Stringent response* (lg2fc: 0.169) (Table S4c).

### Dynamical gene expression patterns of POR and CARB to butyrate challenge

The experimental set-up allowed us to analyze molecular adaptations over time during increasing concentrations of butyrate that enabled insights into dynamics of responses. We categorized RAST subsystems into different groups based on their response pattern consisting of three “converging” (decreasing differences in expression between POR and CARB upon butyrate challenge), three “overshoot” (increased expression at R1 followed by a decrease at R2) and three “diverging” categories (increasing differences in gene expression between POR and CARB upon butyrate challenge) (Figure S4). In line with results of an overall decrease in BCdis between POR and CARB based on gene expression patterns, the majority of subsystems (*n* = 103) were converging between strains of the two groups during butyrate wash-in (Figure S4(a*–*c)), whereas 45 subsystems displayed an “overshoot” mechanism by diverging at R2 and subsequently converging at R3 (Figure S4d, e, f). The category shown in Figure S4(d) comprised several stress response related subsystems that displayed “overshoot” reactions only in POR, such as *Periplasmic acid stress response*, *Oxidative stress response* and *Acid stress response*. Those subsystems were, hence, initially converging with CARB at R2 and only diverged at R3. Furthermore, 53 subsystems followed a “diverging” pattern upon butyrate wash-in increasingly separating the two groups, based on global gene expression, over time (Figure S4(g–i)).

### *Validating results of clinical isolates using a porin-deficient double mutant* E. coli *strain*

**Figure 6.**
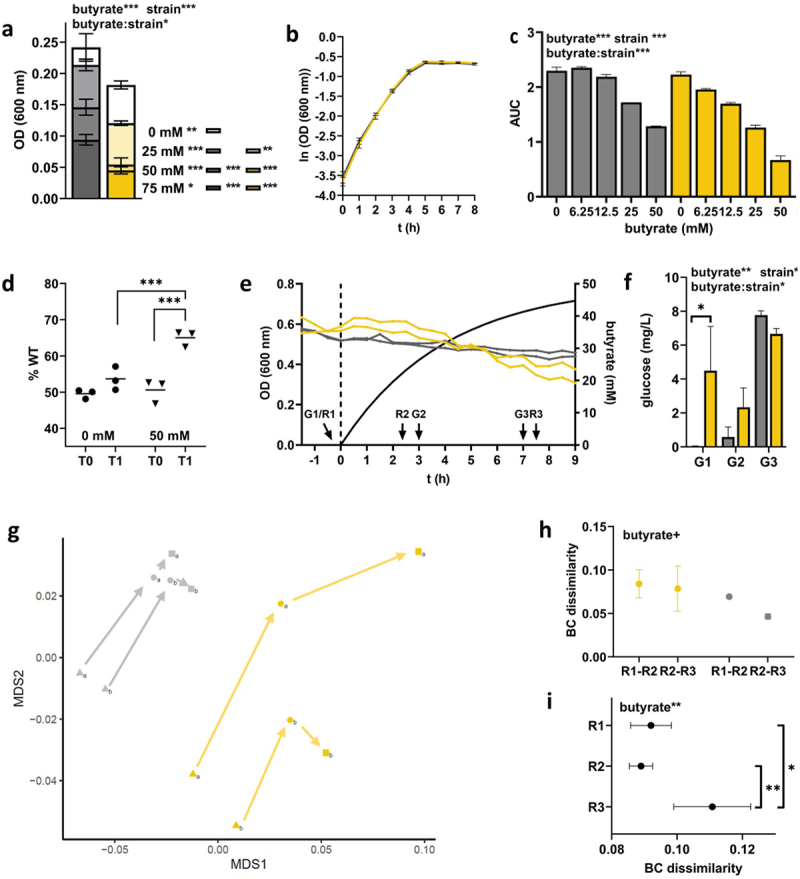
Investigating the growth physiology under anaerobic conditions and responses to butyrate of gut-derived *E. coli* ATCC 8739 (wt; gray) and its *∆ompC/∆ompF* double mutant (mt, yellow). Final batch growth after 48 h (OD 600) at different concentrations of butyrate (0, 25, 50, and 75 mM) is shown in panel a, whereas growth curves at 0 mM butyrate are given in panel b. The influence of butyrate on growth dynamics based on the area under the curve (AUC) calculations from growth curves is displayed in panel c. The mean of replicate samples (*n* = 2) is given, where error bars display standard deviations. Relative abundance of wt (% wt) before (T0) and after (T1, stationary phase) competitive growth between the two strains in batch culture (without (•) and with(▾) butyrate) is displayed in panel d. Results from anaerobic continuous culture experiments in steady-state and during butyrate wash-in is shown in panel e (separated by the dashed line; calculated butyrate concentration is shown as solid black line), whereas the amount of residual glucose concentrations in reactors is displayed in panel f. Global gene-expression analysis of the two strains presented by ordination analysis based on metric multidimensional scaling (MDS) analysis and Bray Curtis dissimilarities (BCdis) is shown in panel g. Results for steady state (0 mM butyrate; triangle; R1) and at butyrate concentrations of 25 mM (dot; R2) and 43 mM (square; R3), respectively, are given. In panel h, BCdis between R1 and R2 and R2 and R3 within strains of the two groups are shown, whereas BCdis between wt and mt at the three time points are given in panel i. Statistical calculations are based on two-way ANOVA analyses with Tukey’s HSD post-hoc testing, where “butyrate” refers to the effect of butyrate, “strain” describes the effect of the double mutation and “butyrate:strain” gives their interaction. +, *, **, ***; *p* < .1, *p* < .05, *p* < .01, *p* < .001.

To verify the role of porin deficiency for general growth physiologies under anaerobic carbon-limited conditions as well as for responses to butyrate challenge a porin-deficient (*∆ompC/∆ompF*) *E. coli* (ATCC 8739) mutant strain (mt) was constructed and compared with the wildtype (wt) strain using the same experimental setups as above for clinical isolates. Results of screening experiments revealed a reduction in OD in both strains with butyrate. In line with POR, mt showed significantly stronger responses decreasing its OD by 70% at 50 mM butyrate concentration (0 mM butyrate: 0.182 ± 0.006; 50 mM: 0.055 ± 0.011), whereas a 40% decrease was observed for wt (0 mM: 0.242 ± 0.022; 50 mM: 0.146 ± 0.013) (Figure 6(a)). No differences in AUC values were observed between mt and wt at 0 mM butyrate (Figure 6(b)). While growth dynamics were unaffected in wt up to a concentration of 12.5 mM butyrate, values continuously declined in mt. Overall, a significantly stronger reduction was observed in mt and at 50 mM butyrate AUC values for mt and wt were 30.04% ± 2.65% and 55.98% ± 1.87%, respectively, compared with results during growth with 0 mM butyrate (Figure 6(c), Figure S5). Direct competition experiments between mt and wt confirmed increased butyrate sensitivity of the porin-deficient strain on growth performance. While the relative abundance of wt and mt remained at equal ratios during competitive growth at 0 mM butyrate, butyrate pressure (50 mM) led to significantly reduced final proportions of the porin-deficient strain (Figure 6(d)).

Next, we cultured the two strains in continuous culture monitoring their response to butyrate wash-in using RNASeq analyses. In steady state (0 mM butyrate), no difference in OD was observed (Figure 6(e)). However, similar to CARB almost no remaining glucose was detected for wt (0.035 ± 0.015 mg L^−1^), whereas significantly higher amounts (4.49 ± 1.85 mg L^−1^) were detected for mt (Figure 6(f)). As seen for clinical isolates during butyrate wash-in, glucose concentrations were slightly increasing for wt reaching similar levels as mt. In line with results for CARB and POR, mt and wt clustered separately based on their gene expression profiles throughout the experiment (Figure 6(g)). Overall, higher responses to butyrate challenge (*p* < .1) were observed from R1 to R2 compared with those from R2 to R3, which was specifically true for wt (Figure 6(h)). Based on BCdis a slight convergence between the two strains after wash-in of butyrate at R2 was detected that was followed by a significant divergence at R3 (Figure 6(i)).

At steady state (0 mM butyrate), detailed analyses of gene expression results revealed many differentially expressed subsystems and genes between mt and wt that were in line with to those observed for POR and CARB (Table 1; Table S4(d)). In particular, as for POR we observed in mt at steady state higher expression of genes coding for the unspecific porins *phoE* (lg2fc: 9.327) and *ompA* (lg2fc: 1.988) as well as higher expression of the subsystem *Stringent response* (lg2fc: 0.217). Furthermore, similar to POR expression of the category *Iron acquisition and metabolism* (lg2fc: 0.321; *fepABCDEG; fhuABCD; feoB*), the *Phosphate ABC transporter* gene (lg2fc: 2.147) and the subsystem *Branched-chain amino acid synthesis* (lg2fc: 0.973) were higher expressed in mt compared with wt. Higher expression levels of the subcategory *Osmotic stress* (lg2fc: 1.619) in mt were also in line with results derived from clinical isolates. Parts of the oxidative stress response, namely, genes coding for *Glutaredoxin 1* (grx; lg2fc: 1.020) and *sod(Fe)* (lg2fc: 0.912) were higher expressed in wt, as observed for CARB. In contrast to clinical isolates, the subsystems *Oxidative stress, Periplasmic acid stress response* and *Universal stress proteins* as well as the gene *copA*, that were all higher expressed in CARB than POR at steady state (0 mM butyrate), showed similar expression levels in mt and wt.

**Table 1.** Major features differentially expressed between POR and CARB at steady state (0 mM butyrate) compared with results obtained from an artificially introduced porin loss (ATCC 8739 wild-type (wt) vs ATCC 8739 *∆ompC/∆ompF* (mt)). Results observed in both systems are highlighted in bold, whereas those only significantly different between POR and CARB are given in standard font.

↑POR (mt)	↑CARB (wt)
**Unspecific porins (*phoE*, *ompA*) Stringent response Iron acquisition and metabolism Phosphate ABC transporter Branched-chain amino acid synthesis Osmotic stress response**	Periplasmic acid stress response Oxidative stress response Universal stress protein family Copper transport

Stronger responses of mt to butyrate in batch culture and its inferiority compared with wt in direct competitive growth verified porin deficiency as a key feature for observed increased sensitivity of POR strains to this gut microbiota-derived metabolite (Figure 6(d/e)). In steady state, results from POR and CARB were largely reproduced in the model system, however, responses in gene expression to butyrate challenge were only partly reflecting results derived from clinical isolates (Table 2; Table S4(e,f)). In line was an upregulation of the oxidative stress related gene for *Glutathionylspermidine synthase* (lg2fc mt: 0.942/lg2fc wt: 1.317), the subsystem *Biotin biosynthesis* (lg2fc mt: 1.434/lg2fc wt: 1.330), the cysteine biosynthesis related *cysA/P* sulfate uptake transporter (lg2fc mt: 1.357/lg2fc wt: 0.710) and the copper transport enzyme (*copA;* lg2fc mt: 1.048/lg2fc wt: 0.324) in both mt and wt. In contrast, *Cycloproprane-fatty acyl phospholipid synthase* (lg2fc: 1.234) and the subsystem *Glutaredoxins* (lg2fc: 0.260) were only increased in wt and not in mt, while expression of the subsystem *Cysteine biosynthesis* as well as the zinc transporter *zitB* did not significantly respond to butyrate in either strain (Table 2). Furthermore, the category *Iron acquisition and metabolism* was only reduced in mt (lg2fc: 1.038) mainly driven by the pathways *efeOUB* and *fepABCDEG*, whereas an increase was observed in wt (lg2fc: 1.534) mainly driven by *fepABCDEG* and *efeUOB*.

**Table 2.** Major features differentially expressed between POR and CARB during butyrate challenge (↓ downregulation/↑ upregulation) compared with results obtained from an artificially introduced porin loss (wt vs mt). Results observed in both systems are highlighted in bold, whereas those only significantly different between POR and CARB are given in standard font; individual regulation patterns for ATCC 8739 wild-type (wt) and ATCC 8739 *∆ompC/∆ompF* (mt) are shown in brackets.

POR and CARB (mt and wt)	POR (mt)	CARB (wt)
↑**Glutahionylspermidine synthase** ↑Cysteine biosynthesis (mt±/wt±) ↓Iron acquisition and metabolism (**mt**↓ /wt↑) ↑**Copper transport** ↑Zinc transport (mt±/wt±) ↑**Biotin biosynthesis**	↑Acid stress response (mt±) ↑Universal stress protein family (mt±) ↑Oxidative stress response (mt±) ↑Periplasmic acid stress response (mt↓) ↓**Branched-chain amino acid synthesis**	↓**Porins (*lamB, ompC****/F*) ↑**Stringent response**

Apart from a downregulation of the subsystem *Branched-chain amino acid synthesis* (lg2fc: −1.077), features found differentially expressed during butyrate challenge specific for POR strains were not observed in mt. In contrast to POR, expression of the subsystems *Acid stress response* and related genes (*glsA, gadABCE, cadAB*), *Universal stress protein family* and *Oxidative stress* did not change with butyrate challenge; the subsystem *Periplasmic acid stress response* (lg2fc: −1.138) along with its related genes *(hdeAB, gadE, slp, hdeD*) was even downregulated (Table 2). We speculate that the increased expression of those systems in POR with butyrate challenge primarily stem from the low expression levels at steady state (0 mM) that were in contrast with results derived from mt, where similar expression levels of those features as in wt (and in CARB) were observed during growth in steady state without butyrate.

In line with results observed in CARB, we found the subsystem *Stringent response* (lg2fc: 0.187) slightly upregulated as a response to butyrate only in wt, along with a downregulation of the porins *ompC* (lg2fc: −0.647) and maltoporin *(lamB)* (lg2fc: −1.216) (Table 2).

### Investigating membrane stability of POR and CARB strains

**Figure 7.**
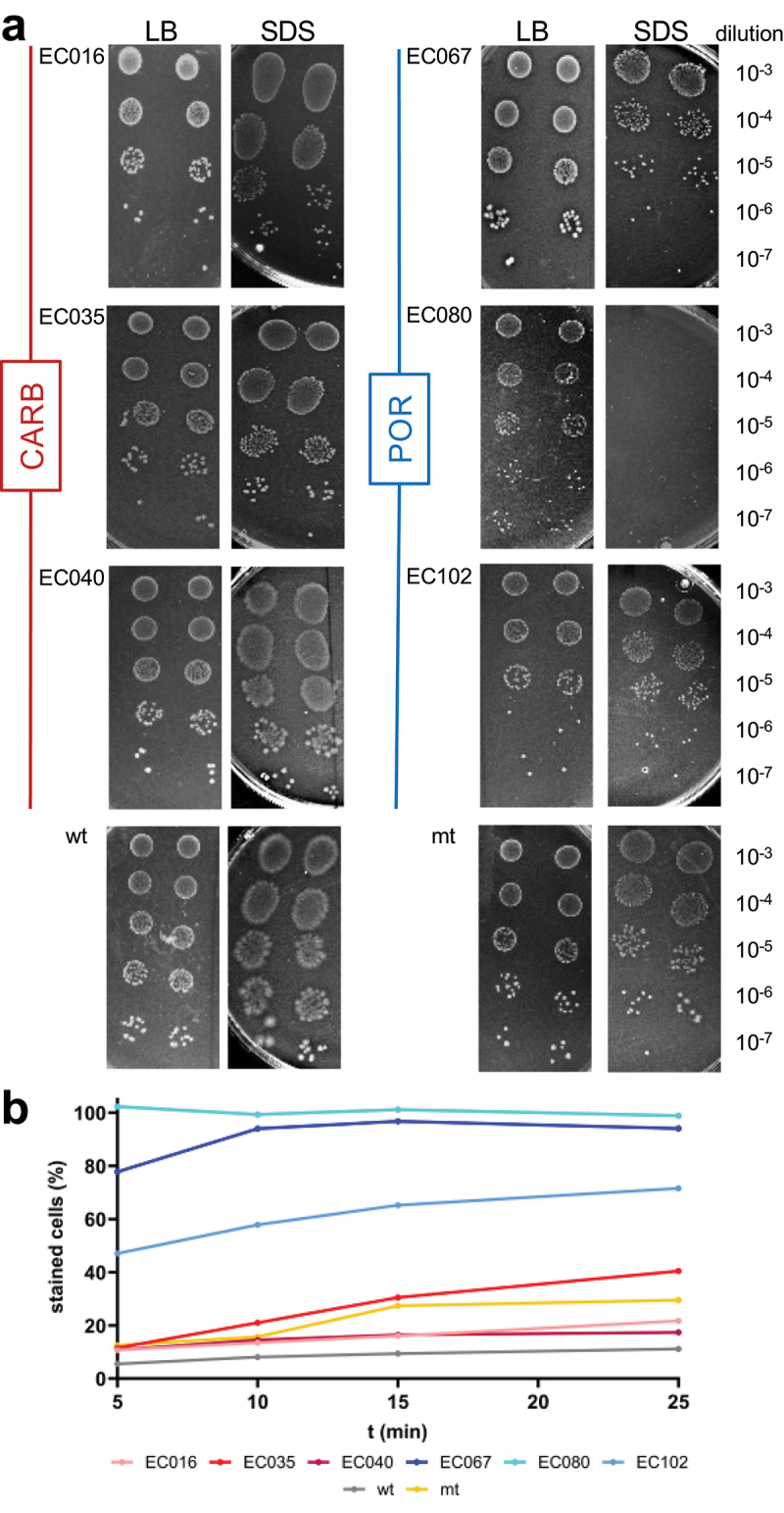
Investigating outer membrane stability of strains. Panel a shows growth on LB agar plates (LB) compared with growth on plates additionally containing 2% of the envelope stressor sodium dodecyl sulfate (SDS); 5 μL of 10^−3^ −10^−7^ dilutions of overnight cultures were spotted in replicates (*n* = 2) on plates and incubated at 37°C under anaerobic conditions. Below (panel b) displays results challenging strains with SDS (0.025%) followed by recording the percentage of SYTOX green positive cells by flow cytometry.

Based on literature and obtained results from this study unspecific porins are expected to play an important role in membrane integrity and we, hence, investigated membrane stability by challenging strains with the envelope stressor SDS (Figure 7). Growth of POR strains were affected by SDS when grown overnight on agar plates, in particular in the case of EC080 and EC067, whereas hardly any responses were seen for CARB strains (Figure 7(a)). We additionally challenged all strains with SDS (0.025%) in liquid culture in the presence of the membrane impermeable dye SYTOX green and followed staining of cells over time. Clear distinctions between the two groups were detected, where POR strains were stained at much higher rates compared with CARB (Figure 7(b); individual results are shown in Figure S6). In line with results from clinical isolates mt responded more to SDS than wt in both experiments, however, mt was much less affected than POR strains.

## Discussion

Our study demonstrates that the resistant mechanism in *E. coli* governs the response to gut microbiota-derived butyrate, where porin-deficient strains were more sensitive to the metabolite compared with those exhibiting a carbapenemase. Results suggest that a decreased stability of the outer membrane and a lower expression of stress response systems in POR were contributing to those observations. It revealed that antibiotic resistance acquisition results in fitness costs that are specific for the resistance mechanism offering opportunities to translate those findings into risk assessment and the development of precision measures to limit the spread of CRE.

OmpC and OmpF function as major pores for uptake of various compounds including sugars, amino acid and ferrous iron (Fe^2+^)^17,34,35^ and their dysfunction is, hence, expected to lead to impaired nutrient intake and growth. Indeed, we observed lower growth yields and reduced growth dynamics (AUC) in POR strains compared with CARB. Our results suggest that malfunctioning of those porins in POR are partly compensated by increased expression of two alternative channels, namely, *ompA* and *phoE* in order to maintain carbon influx,^36^ which was also reproduced in the mutant strain. In our experiments upregulation of *phoE* in POR was primarily driven by one strain (and observed in mt) and we, hence, speculate that rather OmpA is contributing to sustain nutrient influx in clinical isolates.^17,37^ This is in line with reports from Knopp and Andersson demonstrating that specifically a higher expression of *phoE* can lead to loss of resistance by offering new influx routes for antibiotics,^36^ which might be lesser the case for OmpA that represents a narrow-channel porin promoting slow substrate/ion diffusion.^17^ Despite increasingly expressing alternative porins, clinical isolates showed growth deficiencies compared with CARB, even at 0 mM butyrate, indicating fitness disadvantages. This was not seen in mt that performed equal to wt in batch culture at 0 mM butyrate. However, in continuous culture during steady state residual glucose concentrations were higher in mt (compared with wt) and reflected discrepancies as observed between POR and CARB suggesting that substrate affinities are probably hampered in porin deficient strains.^29^ In line with a lower substrate intake-rate, we observed the *Stringent response*, which is known to be induced by various starvations, such as carbon, amino acid and iron,^38,39^ expressed at higher levels in POR (and the mt). Concurringly, we found increased expression of several iron uptake systems and genes associated with the synthesis of branched-chain amino acids, whose levels are sensed by the stringent response system monitoring low intracellular nutrient availability.^40^ In summary, the observed impaired nutrient intake and altered growth properties in POR probably result in major disadvantages when colonizing the human gut, where successful competition for nutrients with gut microbiota is a major feature governing fitness.

Next to occupying nutritional niches, gut microbiota provides colonization resistance against pathogens via the production of metabolites, such as SCFAs and bile acids, as well as secretion of bacteriocins.^19^ It is known that SCFAs prevent growth of gram negatives at acidic pH^41^ and detailed investigations on ampicillin-resistant *E. coli* revealed intracellular acidification as the underlying mechanism.^20^ Among all SCFAs the production of butyrate is a hallmark of a well-functioning gut microbiota^21,42^ and it was, hence, chosen for experiments in this study. Butyrate is known to cause osmotic stress in the extracellular space and to enter the periplasm in its deprotonated form through porins.^43^ We indeed observed downregulation of *ompF* and *ompC* genes along with maltoporin (*lamB)* in all CARB strains. Genes encoding the major *omp* regulators *envZ/ompR* were downregulated as well, however, a causative role for *omp* downregulation cannot be drawn as their phosphorylating state is key for activity and various other pathways are possibly involved in *omp* regulation as well.^44^ Initially, we were hypothesizing porin-deficient bacteria to be more resistant to butyrate challenge due to reduced influx of the metabolite. However, porin malfunctioning does not imply exclusion of butyrate, as its protonated and uncharged form can diffuse through the outer membrane and acidify the periplasm by deprotonating.^19^ Our results suggest this route as the primary mechanism of action, which is supported by the fact that growth of gram negatives in presence of SCFAs is particularly hampered at acidic pH, which promotes the protonated state of butyrate.^20,45^ Our experiments were conducted at mildly acidic pH as observed in the human gut environment. Minor differences in pH values between continuous cultures, with on average slightly lower values for CARB strains, were recorded. However, given that this group reacted even less to butyrate we do not consider pH as a potential source for biasing observed results. Next to intracellular acidification butyrate acts by intercalating in the inner membrane of bacteria increasing its fluidity. Upregulation of *cfa (cycloproprane-fatty acyl phospholipid synthase)* that increases rigidity of the inner membrane by integration of unsaturated fatty acids^43^ along with *Biotin biosynthesis*, a fatty-acid related vitamin involved in production of membrane lipids,^46^ was observed in all isolates and an increased membrane turnover during butyrate challenge can, hence, be considered as a general response of *E. coli* in order to sustain stability and homeostasis. Concerning the outer membrane, porin deficiency is known to affect its stability where *ompC* plays a key role for rearrangement of phospholipids maintaining lipid asymmetry and membrane homeostasis.^18^ Along this line our experiments demonstrated a higher sensitivity to the membrane stressor SDS in POR compared with CARB, which is further supported by an observed increased osmotic stress response at steady state by former strains. Thus, our results indicate that impaired integrity of the outer membrane of porin-deficient strains is a contributing feature governing their high sensitivity to butyrate, probably due to increased influx of the metabolite. Those observations were also reflected in mt that reacted more to SDS and showed higher expression of osmotic stress response genes at steady state compared with wt, however, extents were much less compared with POR strains. A previous study showed similar effects of SDS on envelope stress in an *∆ompC/∆ompF* K12 MG1655 strain.^16^ We were initially conducting experiments based on this model strain, but realized that it reacted very sensitive to butyrate, at similar magnitudes as POR and its corresponding *∆ompC/∆ompF* mutant did hardly grow at concentration of 25 mM butyrate in screening experiments (data not shown). We, hence, specifically chose a strain that was isolated from the gut and that was able to reflect eco-physiological properties required in *in situ* conditions over the laboratory working horse K12 MG1655.

A major discriminator between POR and CARB during steady state growth before butyrate challenge were lower expression of genes associated with various stress responses in porin-deficient strains involving the *Periplasmic acid stress response*, *Acid stress response*, *Universal stress protein family* and *Oxidative stress*. Upon butyrate challenge all four subsystems were strongly upregulated that was accompanied by increased expression of genes associated with the general stress response, which was also observed in CARB. Detailed analyses revealed an “overshoot” expression of those subsystems in POR, where an initial upregulation from R1 to R2 followed a slight downregulation from R2 to R3 was detected. However, expression levels in POR remained below those of CARB during the entire experiment indicating impaired stress responses (except for osmotic stress response) in those strains probably contributing to the higher sensitivity of POR strains to butyrate hampering their abilities to take counter measures against (increased) influx of the SCFA.

An impaired stress response was not reproduced in the mt strain that showed higher expression of associated genes similar to that of the wt and CARB strains. We speculate that this discrepancy is due to the fact that naturally acquired porin deficiency in clinical isolates promoted downstream evolutionary events selecting for features that maintain sufficient nutrient supply, which are in trade-off with properties important for survival, including stress response mechanisms, as described in the model of SPANC (self-preservation and nutrient competition) balance.^13^ In contrast, porin deficiency in mt was artificially introduced on the background of a well-functioning stress response that was still expressed at normal levels. Within this study gene content and SNP analyses did not reveal any signatures for adaptive evolution of POR counteracting porinmalfunctioning in the context of the SPANC theory and investigations including more strains coupled to *in vitro* and *in vivo* evolution experiments under various conditions will be performed in order to shed light on this topic. Additional insights using (spatial) proteomics and metabolomics will further assist those efforts. Furthermore, in mt complete *omp* genes were deleted, whereas clinical isolates did still contain respective gene sequences. Those were probably largely functionally impaired due to fragmentation/interruption, however, certain remaining functionalities of those gene products cannot be completely ruled out.

In this study, we showed a direct effect of butyrate on antibiotic resistant bacteria, however, *in vivo* this metabolite acts on multiple levels. Next to directly interfering with bacterial growth butyrate promotes barrier integrity by feeding epithelial cells and promoting mucus production and acts anti-inflammatory.^47^ This concert of action is probably key to prevent both colonization and infection with harmful bacteria and reduced levels of this metabolite have been revealed as a major clinical risk factor for colonization and infection with CRE.^5^ In line with those observations initial trials based on fecal microbiota transplantation have proven partly successful for CRE decolonization.^48^ Results from our study suggest that such measures are particularly effective for porin deficient CRE given their fitness disadvantages. Results from competition experiment, where mt lost against wt under butyrate pressure, substantiates this hypothesis. It should also be mentioned that under specific circumstances porin deficiencies might also lead to increased fitness, such as increased resistance against macrophages^49^ and evasion of phages that often use porins as target sites.^50^ In any case, our study emphasizes the importance to stratify antibiotic-resistant bacteria based on their resistance mechanisms in order to develop multi-level precision measures, also involving gut microbiota, to limit the spread of and infection with those bacteria in a targeted, personalized manner.

## Supplementary Material

Supplemental Material

## Data Availability

Raw sequencing data is available at the European Nucleotide Archive (PRJEB74475).
