# Supplementary material for: Gut microbiota-derived butyrate selectively interferes with growth of carbapenem-resistant Escherichia coli based on their resistance mechanism
Source: Gut Microbes. 2024 Sep 18;16(1):2397058. doi: 10.1080/19490976.2024.2397058 (PMC11529417; doi:10.1080/19490976.2024.2397058)
Supplement: Supplemental Material [file KGMI_A_2397058_SM0848.zip › Supplementary files 2.docx]

**Figure S1.** Overview of all clinical isolates (POR n=15, blue; CARB n=15, red) and ATCC 8730 (black) used in the study. On panel a on the left a dendrogram based on average nucleotide identity is given indicating the resistance mechanism (POR (blue) and CARB (red)). Type of carbapenemases, integrity of genes encoding OmpF and OmpC, and detected extended-spectrum lactamases (ESBL) based on *in silico* analyses as well as measured MIC values based on VITEK 2 are given in the table on the right. Panel b and c show the protein alignments of *ompC* and *ompF* genes, respectively, for the six selected strains (three CARB and three POR) as well as for the model strain ATCC 8739 used for detailed experiments.

**Figure S2.** Pangenome and single nucleotide polymorphism (SNP) analyses of POR and CARB strains. Panel a shows orthologous genes categorized based on different occurrences between the two groups, where “carb_por” refers to the same occurrence in both groups, whereas genes found more often in POR strains are shown on the right and those more frequently detected in CARB are displayed on the left. For instance, the category “por5” harbors all genes that were detected in five more POR than CARB strains. In panel b results based on the same categories for SNPs using ATCC 8739 as the reference are given. For both analyses no obvious features were discovered clearly differentiating groups – most features were observed in similar amounts of strains of each group and none were recorded in the flanking categories.

**Figure S3.** Growth curves of all clinical isolates (CARB, red; POR, blue) in presence of different butyrate concentrations (0 mM, 6.25 mM, 12.5 mM 25 mM and 50 mM).


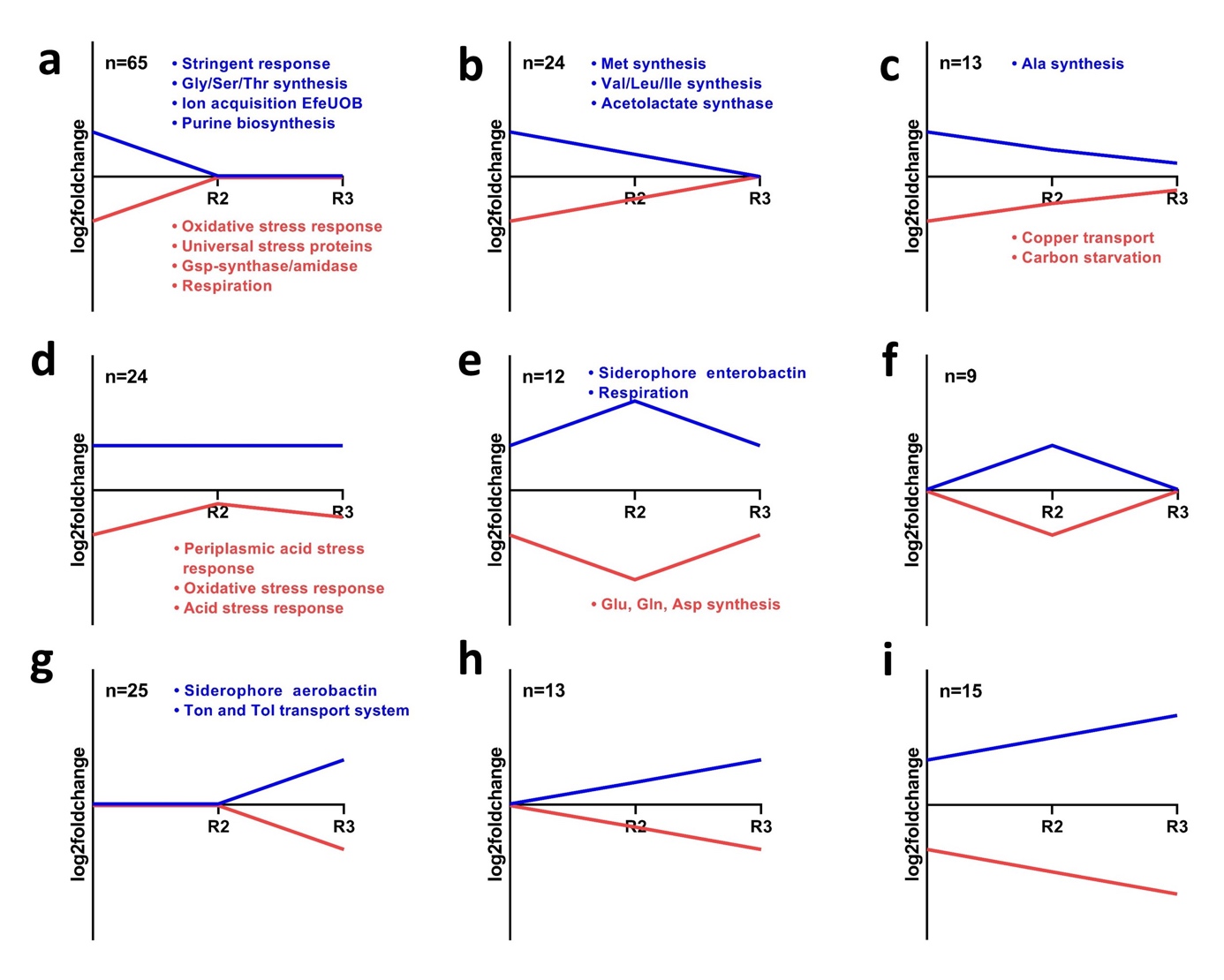


**Figure S4.** Significantly differentially regulated RAST subsystems binned by their regulation pattern upon butyrate challenge based on log2foldchange at steady state (R1, 0 mM butyrate), R2 (25 mM butyrate) and R3 (43 mM butyrate). Examples for subsystems belonging to individual groups are given (blue, higher expressed in POR; red, higher expressed in CARB). Patterns representing converging regulation are given in panel a, b and c, while panel e and f signify initial “overshoot” regulation. Panel d can be also be attributed to this pattern as conversion at R2 is mainly driven by upregulation in POR converging with CARB; however, expression was consistently higher in CARB throughout the experiment. Patterns representing a diverging regulation are displayed in panel g, h and i.

**Figure S5.** Growth curves of *E. coli* ATCC 8739 (wt; grey) and its *∆ompC/∆ompF* double mutant (mt, yellow) in presence of different butyrate concentrations (0 mM, 6.25 mM, 12.5 mM 25 mM and 50 mM).

**Figure S6.** Flow cytometric biplots (sideward-scatter (SSC) vs green fluorescence (B1A)) of individual cultures challenged with SDS in the presence of SYTOX green measured after different incubation times (5 min, 10 min, 15 min, and 25 min). Results from SYBR Green I staining are shown as well.

**Table S1.** Oligonucleotides used in this study.

| **Primer** | **Sequence** | **Reference** |
| --- | --- | --- |
| ompC-P2 | TGCAGTGGCATAAAAAAGCAAATAAAGGCATATAACAGAGGGTTAATAACCATGGTCCATATGAATATCCTCCTTA | This study |
| ompC-P0 | AAAACAATGAAAAAAGGGCCCGCAGGCCCTTTGTTCGATATCAATCGAGA ATTGTGTAGGCTGGAGCTGCTTC | This study |
| ompF-P2 | ATTGACGGCAGTGGCAGGTGTCATAAAAAAAACCATGAGGGTAATAAATACATGGTCCATATGAATATCCTCCTTA | This study |
| ompF-P0 | AAACAGGACCAAAGTCCTGTTTTTTCGGCATTTAACAAAGAGGTGTGCTAATTGTGTAGGCTGGAGCTGCTTC | This study |

**Table S2.** Monitoring of pH during growth of strains in continuous culture.

**Table S3.** Detailed results of significantly differentially expressed key features between strains of the two groups during growth in steady state at 0 mM butyrate that are shown in Figure 4 are given. The average TPM values for all strains are given. Features are categorized based on higher expressed in POR (blue) and CARB (red), respectively, where dark colors refer to higher expressed in that strain compared to all three strains of the other group and lighter shades signify higher expression compared with two strains of the other group.

**Table S4.** List of all significantly differentially expressed genes based on all shared genes in POR and CARB as well as for ATCC 8739.

**Table S5.** Detailed results of significantly differentially expressed key features as a response to butyrate challenge that are shown in Figure 5 are given. Features significantly regulated in both groups (top) and only in POR (bottom) are shown. Color codes refer to significantly increased (green) and decreased (gold) expression upon butyrate challenge based on results of each individual strain, where respective lighter shades indicate (non-significant) responses.
